# Progress in Surgery

**Published:** 1902-06-14

**Authors:** 


### Progress in Surgery.

(*Continued from page* 169.)

### OTOLOGY.

**Extreme Deafness and Tachycardia cured by Cessation of Smoking.**—Douglas Stanley[3] relates a case of this kind. The patient was a soldier who had been in service in South Africa. The deafness had come on about a month after the patient's discharge from a hospital in which he had been detained for typhoid fever. In two or three days the deafness became extreme. His pulse was 144 when seen a week or two later by Stanley, and had been even more frequent for a time previously. The man had been smoking eight or nine ounces of very strong twist per week for several weeks. The smoking was stopped, and when the patient was seen a month later the pulse was 90 and of much better quality, and the deafness had practically disappeared. He had not had any trouble with his sight. The case looks as if it had been one of toxic deafness similar to tobacco ambliopia.

**Otorrhœa and its Treatment.**—A. G. Cumberbatch[4] discussed this subject in a clinical lecture. The following points were brought out. Running from the ear is, after sclerosing catarrh, the commonest form of ear disease. It is only comparatively recently that insurance companies have refused to insure the lives of patients thus afflicted, or only agreed to do so for an increased premium. Chronic discharge is nearly always the result of imperfect treatment of acute otitis. Being much more common after scarlet fever and measles than after ordinary naso-pharyngeal catarrh, it is much more frequently seen in early life. Even adults often date the trouble from childhood, though years of quiescence may have intervened. The remains of the membrane are generally thickened, sodden, and whitish if there is much discharge. Often the meatus is narrowed by subdermic thickening. If no perforation can be seen the discharge may be proved to come from the tympanum by the detection of mucus in it which is insoluble in water. No positive prognosis can be given as to the result of any treatment short of operative interference, either as to the discharge or the hearing power. For syringing lotions of carbolic acid, biniodide or perchloride of mercury, formalin, lysol, chinosol, and lastly, tachiol may be used. To remove crusts and epithelial débris first soften them in a solution of sodium bicarbonate, introducing it at night and syringing in the morning, and continue this until the ear is quite free from them. The author has not found peroxide of hydrogen superior to the lotions mentioned, but it may be tried if the discharge is very foul. After cessation of discharge the perforation more often remains open than closes. The non-closure depends largely on the size and the callousness of the edges of the opening. Unless large, an opening may be sometimes made to close by irritating its edges with some caustic, and of the caustics perhaps chloracetic acid is the best. The ear must be syringed immediately after its use, as the acid is very deliquescent It is wiser not to attempt to close the aperture if the hearing is good. If the above treatment fails it may be due to the perforation being very small, in which case it should be enlarged. If the perforation is large and the mucous membrane hypertrophied and highly vascular, alcohol may be tried, or failing that a free application of chromic acid, or of the galvano-cautery; or if the malleus and incus are destroyed, curetting the tympanic cavity. Gauze packing after this is often successful. Discharge may persist, with a good opening present and without much change in the membrane, in which case either an ossicle or some part of the tympanic wall is carious. For the former ossiculectomy is the treatment. A very fibrous polypus can be expeditiously removed with a torsion forceps. Max Toeplitz [5] writes on otorrhœa in children. At birth the mastoid process contains one large cell, from which the other mastoid cells develop towards the apex, until in the third year the process is almost constructed like that of an adult. The formation of a cavity in the tympanum takes place not later than the first day of life. The suppuration in the ears of infants is due to infection through the Eustachian tube. In the acute form the temperature may be 104°, and vomiting, stupor, delirium, or convulsions be symptoms. Thus meningitis and "dental convulsions" may be closely simulated. There is a latent form of the affection in which restlessness, rise of temperature, and loss of weight may be absent. In periostitis of the mastoid the extension takes place through the mastoideo-squamous suture. The otitis of measles begins quite early. It runs a milder course than that in scarlatina. In influenza there is a marked tendency to hæmorrhage into the ear and to implication of the mastoid. In pneumonia otitis occurs during the second or third week. The most common bacteria which cause suppuration are the diplococcus pneumoniæ, and the streptococcus and staphylococcus pyogenes. These are all normally present in the nose, naso-pharynx, and mouth, but do not act pathologically until the resistance of the body is weakened. Hartmann found that 75 per cent. of infants had otitis, and in children otitis is twice as common as in adults. The nose and throat cause 35 per cent. of these, and adenoids 7½ per cent. The ears of 20 per cent. of school children were found to be diseased. As soon as one is convinced in acute cases that there is secretion in the tympanic cavity, paracentesis should be performed in the lower posterior segment, after thoroughly sterilising the meatus with 1 in 4,000 corrosive sublimate. Dry treatment is better than that with the syringe, but cannot be used for hospital out-patients. The examination of the ear of an infant is difficult. The walls of the meatus meet and the membrane is nearly horizontal. One has to clear the meatus with forceps. A long small speculum must be used. The drum is nearly as large as in the adult, *i.e.*, 9 mm. in diameter, but it is seen at a different angle and the malleus appears shorter. The drum does not bulge with the suppuration. Hellat [6] states that purulent otitis is less severe in Russia than in Western Europe. He recommends cleaning the middle ear by the dry method, and uses small swabs of cotton-wool twisted upon the ends of wooden holders; after use the end of the stick is broken and fresh wool applied. Finally dry boracic powder is blown into the ear. Syringing is necessary before drying if the pus is very thick. If the radical mastoid operation is performed the writer does not recommend any plastic operation on the cartilaginous meatus.

[3] Birmingham Med. Rev., Dec. 1901, p. 355. [4] Clin. Jour., March 26, 1902, p. 353. [5] Post-Graduate, Feb. 1902, p. 189. [6] Brit. Med. Jour. Epitome, Feb. 1, 1902.

